# Propofol Infusion Is a Feasible Bridge to Extubation in General Pediatric Intensive Care Unit

**DOI:** 10.3389/fped.2020.00255

**Published:** 2020-05-28

**Authors:** Utpal S. Bhalala, Abhishek Patel, Malarvizhi Thangavelu, Morris Sauter, Elumalai Appachi

**Affiliations:** ^1^The Children's Hospital of San Antonio, San Antonio, TX, United States; ^2^Baylor College of Medicine, Houston, TX, United States

**Keywords:** propofol, sedation, mechanical ventilation, extubation, children, respiratory failure

## Abstract

**Objective:** The current literature on propofol infusion as a bridge to extubation in critically ill children is limited to children with burns and congenital cardiac disease. We hypothesize that propofol infusion is a feasible bridge to extubation in mechanically ventilated, critically ill children.

**Design:** Retrospective chart review.

**Setting:** Pediatric intensive care unit of a tertiary care teaching hospital.

**Patients:** Children < 21 years, admitted to our Pediatric intensive care unit (PICU), requiring mechanical ventilation (MV) for at least 48 h and at least two sedative infusions and who received propofol infusion for 4 to 24 h during anticipated extubation from January 2014 to May 2017.

**Interventions:** None.

**Measurements and Main Results:** We assessed extubation success as primary outcome. We defined extubation success as no re-intubation within 24 h after extubation. We also assessed for occurrence of adverse effects of propofol infusion (1) hemodynamic instability [more than 10% change from pre-propofol baseline heart rate (HR) and mean arterial pressure (MAP) measured 4 h before and during propofol infusion, need for any inotrope and/or fluid bolus] and (2) occurrence of lactic acidosis in absence of any documented sepsis. We compared hemodynamic parameters before and during infusion using Wilcoxon Rank Sum Test (significant *p*-value ≤ 0.05). We evaluated 35 critically ill, mechanically ventilated children. The median age, weight and duration of MV were 3.8 (IQR: 1.25–10.5) years, 12 (IQR: 6–16.2) kilograms and 111 (IQR: 78–212) h, respectively. Of the 35 patients, 15 (43%) were post-surgical (10 general and 5 cardiac) and the remaining 20 (57%) were non-surgical respiratory failure cases. The median (IQR) propofol infusion dose and duration were 64.7 (53.2-81.1) mcg/kg/min and 7.8 h respectively. Only one patient got re-intubated within 24 h of extubation and was later diagnosed with vascular ring. During propofol infusion, 7/35 (20%) patients exhibited transient drop in MAP > 10% from baseline, but none had lactic acidosis or required an inotrope or fluid bolus.

**Conclusions:** In critically ill, mechanically ventilated patients, propofol infusion used over a short duration (<12 h) was found to be a feasible bridge to extubation. No patient had significant hypotension or lactic acidosis during the infusion.

## Introduction

Extubation of critically ill children who have been maintained on large doses of sedatives is challenging. When they are close to an extubation trial, balancing the need for optimal sedation to prevent accidental extubation due to agitation and a need for optimum respiratory drive and airway reflexes for a successful extubation is crucial (1, 2). The weaning of sedation during transition to extubation is often not smooth, thereby delaying the extubation (3). The common sedatives used for intubated, mechanically ventilated children are opioids, benzodiazepines and dexmedetomidine. The opioids and benzodiazepines are associated with tolerance, need for higher doses to achieve good sedation level over time and tendency to accumulate in lipid stores of body. Dexmedetomidine, a short acting sedative has been described to facilitate extubation in critically ill adult patients (4, 5). Dexmedetomidine used as a peri-extubation sedative has been shown to facilitate ventilator weaning in critically ill children (6). In critically ill children after surgery for congenital heart defects, Dexmedetomidine was not associated with success of early extubation and was associated with significantly higher rescue sedatives than the control (7). Overall, there is a conflicting data regarding use of dexmedetomidine to facilitate extubation trial in critically ill children and has been reported to be associated with severe bradycardia, cardiovascular instability, and cardiac arrest in children (8). Propofol is a short-acting medication that works through GABA receptor to facilitate decreased level of consciousness and lack of memory for events (9). There were 2 previous pediatric studies regarding propofol as a bridge to extubation, but they focused on the special population—children with burns and children with the congenital cardiac disease after the cardiac surgery (10, 11). A survey study conducted in Germany showed that 30% of the pediatric intensive care units (PICUs) in Germany use propofol as a bridge to difficult extubation (12). Even though propofol infusion is used as a bridge to extubation, there is a very limited data in the pediatric population regarding propofol infusion for smooth and successful extubation, especially in critically ill children supported on mechanical ventilation for at least 48 h. We performed a retrospective chart review to assess the use of propofol infusion for a successful extubation in critically ill children who required mechanical ventilation for at least 48 h in our general PICU. To our knowledge, this is the first study, which explored feasibility of propofol infusion as a bridge to successful extubation in a general PICU.

## Materials and Methods

### Study Design and Selection of Participants

The study was conducted in the general PICU at The Children's Hospital of San Antonio (CHofSA), a freestanding, 200-bed, tertiary care children's hospital with more than 1,400 PICU admissions annually. Baylor College of Medicine institutional review board and CHofSA feasibility committee approved the study. Due to retrospective nature of the study, our IRB approved the study with a waiver of informed consent.

The study was a retrospective chart review of children <21 years, admitted to our PICU requiring mechanical ventilation (MV) for at least 48 h and who received propofol infusion for 4 to 24 h during anticipated extubation between January 2014 to May 2017.

Our primary outcome was extubation success defined as no re-intubation within 24 h after extubation. Our secondary outcomes included propofol-induced hemodynamic instability and occurrence of lactic acidosis in absence of any documented sepsis. The criteria used in our study to define propofol-related hemodynamic instability were adopted from a previous pediatric study—more than 10% increase in heart rate (HR) with more than 10% decrease in mean arterial pressure (MAP) as compared to pre-propofol baseline measured 4 h before and during propofol infusion and need for any inotrope and/or fluid bolus during propofol infusion (11). In our unit, point of care blood gas analysis, which is a routine blood gas analysis, also provides lactate values. In our unit, all the patients get periodic point-of-care blood gas analysis, including lactate levels during weaning of the ventilator support, during CPAP trial, and prior to extubation.

### Inclusion Criteria

1) All critically ill children <21 years of age intubated and mechanically ventilated for > 48 h in our PICU and on ≥ 2 sedative infusions
2) Use of propofol infusion for > 4 h during extubation trial.

### Exclusion Criteria

1) Received propofol infusion but not extubated due to underlying pathology
2) Patients who received only propofol boluses.

Explanation for including critically ill children intubated and mechanically ventilated for > 48 h in our PICU and on ≥ 2 sedative infusions—In our unit, in absence of any contraindication, propofol infusion is routinely used as a sedative for MRI. In such scenarios, it is a routine practice to run propofol infusion between 1 and 4 h and then discontinue the infusion after the completion of the MRI, and extubate the patient if extubation was deemed appropriate.

Typically, critically ill children who are mechanically ventilated for several days tend to develop tolerance to routinely used sedatives and therefore need larger infusion doses of two or more sedatives. These patients pose significant challenges while trying to balance sedation and ventilator wean while getting them ready for extubation and keeping them safe. Since our primary aim was to evaluate propofol as a bridge to extubation among critically ill, ventilated patients who were maintained on good sedation infusions, we included patients who were on ventilator for at least 48 h and who needed at least 2 sedation infusions before propofol infusion.

Statistical analysis was performed using State version 12 (StatCorp LLC, College Station, TX, USA). The parameters were expressed as median (IQR) and the proportions were expressed as percentages. The median (IQR) hemodynamic parameters and lactate levels before and during propofol infusion were compared using Wilcoxon Rank Sum Test (significant *p*-value ≤ 0.05).

## Results

**Figure 1:**
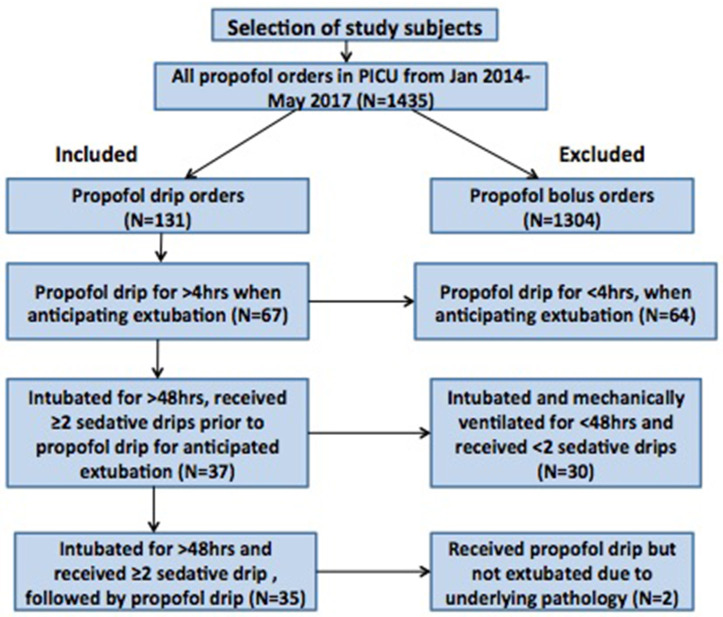
Flow diagram explaining patients included and excluded in the study.

Between January 2014 and May 2017, our pharmacy reported a total 1,435 propofol orders from the PICU. Out of these, 1304 propofol orders were propofol bolus orders and only 131 orders were propofol infusion orders. None of the patients in our study had propofol infusion for >24 h during the study period. Out of the 131 cases that had propofol infusion orders, 67 cases had propofol infusion used for >4 h around the time of extubation. Ultimately, out of these 67 cases, a total 35 critically ill children were intubated for > 48 h and sedated with at least 2 sedative infusions before initiation of propofol infusion around extubation (Figure 1). The median age [median (IQR)] of the patients was 3.8 (1.25–10.5) years. There were 43% patients who were under the category of surgical diagnosis and the remaining 57% under the category of non-surgical diagnosis (Table 1). The median (IQR) duration of mechanical ventilation was 111 (78–212) h. In our cohort, 22/35 (63%) patients had underlying comorbidities such as chronic lung disease, global developmental delay, congenital heart disease, bronchial asthma, underlying genetic or metabolic disease. None of the patients had previous failed extubation attempt. Though due to retrospective nature of the study, we do not have all the details of severity of illness, the duration of ventilation [median (IQR): 111 (78-212) h] and the comorbidities in the study cohort is representative of our overall PICU population.

**Table 1:** Demographic and clinical details (*N* = 35).

**Parameters**	**Observed values, Median (IQR)**
Age (Years)	3.8 (1.25-10.5)
Sex	F = 16 (46%) & M = 19 (54%)
Weight (kilograms)	12 (6-16.2)
**Diagnosis**	
Respiratory failure following RTI	13
Post-cardiac surgery	4
Post-surgical	11
Upper airway obstruction	3
“Unable to protect the airway due to AMS”	1
“Acute respiratory failure following acute decompensation”	3
Surgical	16(46%)
Non-surgical	19(54%)

*F, Female; M, Male; RTI, Respiratory Tract Infection; AMS, Altered Mental Status*.

The median (IQR) propofol infusion dose and duration were 64.7 (53.2–81.1) mcg/kg/min and 7:54 (6:25–11:39) h, respectively (Table 2). In this cohort, morphine and dexmedetomidine infusions were the most common sedation infusions used [31/35 (88%) and 34/35 (97%) patients, respectively]. Hydromorphone, fentanyl, midazolam and ketamine infusions were used during PICU course in 2/35 (5%), 4/35 (11%), 16/35 (45%), and 1/35 (2%) patients, respectively. Around the clock and as needed lorazepam was used in 32/35 (91%) patients. The median (IQR) starting dose and peak dose for propofol infusion were 50 (27.5–55) and 75 (67.5–100) mcg/kg/min, respectively. In 18/35 (51%) patients, propofol infusion was used as the only sedation agent around the time of extubation. In the remaining patients, other sedatives such as around the clock methadone, lorazepam and/or dexmedetomidine infusion were continued with propofol infusion. The propofol infusion was stopped just around the time of extubation in all the patients. During propofol infusion, 7/35 (20%) patients had drop in MAP >10% from baseline, but none required inotrope or fluid bolus. All the patients included in the analysis had periodic point-of-care blood gas analysis, including lactate levels during weaning of the ventilator, during CPAP trial, and around extubation, while they were on propofol infusion. We therefore believe that no case of lactic acidosis was missed. There was no significant difference in median (IQR) lactate levels before [1.21 (0.97–1.64) mmol/L] and during [1.44 (1.13–1.66) mmol/L] propofol infusion (*p* = 0.19). None of the patients developed a significant lactic acidosis (> 2 mmol/L) during propofol infusion.

**Table 2:** Details of propofol, non-propofol sedatives, muscle relaxants used in our cohort and details of intubation and extubation in our cohort (*N* = 35).

**Parameter**	**Observed values, Median (IQR)**
**Propofol details**	
Total dose of propofol (mg/kg)	32.46 (18.86–54.73)
Total dose of propofol (mcg/kg/min)	64.7 (53.2–81.1)
Starting dose of propofol infusion (mcg/kg/min)	50 (27.5–55)
Peak dose of propofol infusion (mcg/kg/min)	75 (67.5–100)
Dose of propofol during extubation (mcg/kg/min)	50 (5-93.75)
Duration of propofol infusion (H:M:S)	7:53:30 (6:24:30–11:38:30)
**Pre-Propofol cumulative dose of sedatives and muscle relaxant**	
Cumulative dose of Morphine (mg/kg)	10.34 (5.15–25.57)
Cumulative dose of Hydromorphone (mg/kg)	0.96 (0.6–1.32)
Cumulative dose of Fentanyl (mg/kg)	4 (2.11–135.75)
Cumulative dose of Dexmedetomidine (mcg/kg)	54.08 (26.53–112.65)
Cumulative dose of Midazolam (mg/kg)	3.23 (1.61–11.09)
Cumulative dose of Lorazepam (mg/kg)	1.55 (0.73–5.29)
Cumulative dose of Cis-atracurium (mg/kg)	6.1 (5.4–11.4)
Cumulative dose of Vecuronium (mg/kg)	1.85 (0.54–12.32)
Cumulative dose of Rocuronium (mg/kg)	7.12 (3.05–12.16)
Cumulative dose of Ketamine (mg/kg)	3.5 (2.5–12)
**Post-extubation sedatives (first 48 h post-extubation)**	
Total dose of Morphine (mg/kg)	0.21 (0.1–0.5)
Total dose of Dexmedetomidine (mcg/kg)	12.4 (9.48–25.88)
Total dose of Lorazepam (mg/kg)	0.52(0.25–1.05)
**Intubation and extubation details**	
Total duration of intubation (Days)	6 (5–11)
Successfully extubated	35 (100%)
Reintubated within 24 h	1 (3%)

A successful extubation was seen in 97% of patients who received propofol infusion as a bridge to extubation. Dexmedetomidine infusion was either continued or re-started within 24 h of extubation in 17/35 (48%) of patients. In only 16/35 (45%) patients, withdrawal assessment tool −1 (WAT-1) scores were available after extubation and in 12/16 (75%) patients; withdrawal to narcotic and/or opioid was documented with a WAT-1 score of ≥ 3 after extubation. One patient got re-intubated within 24 h of an extubation attempt following use of propofol infusion. This patient was found to have failed extubation attempts due to severe upper airway obstruction due to a vascular ring.

## Discussion

Our findings suggest that propofol is a feasible option as a peri-extubation sedative agent in critically ill children supported on mechanical ventilation in general pediatric critical care unit. The prior studies in children have looked at propofol infusion as a bridge to extubation among critically ill children with burns and congenital heart defects. Our cohort included all the critically ill patients who had mechanical ventilator support for at least 48 h, irrespective of the underlying cause of respiratory failure. In short, this is the first retrospective chart review which assessed feasibility of peri-extubation propofol infusion in all critically ill children irrespective of underlying pathophysiology. In the study by Teng et al. all the patients were successfully extubated, whereas in the study by Sheridan et al. 82% patients had successful extubation. In our study, the successful extubation on propofol infusion was found to be 97%—between what was reported by the prior studies. Successful extubation of critically ill children is challenging. Providing appropriate sedation to critically ill, intubated and mechanically ventilated children is imperative. During their recovery from acute cardiorespiratory compromise, the intensive care team is poised with a challenge of weaning sedatives in order to allow spontaneous breathing and also minimize patient agitation and accidental extubation and/or removal of important lines and tubes. Weaning sedatives around the time of extubation and keeping the lines and tubes in appropriate position is often a tough balance. It is especially challenging in those patients who develop tolerance to sedatives. In patients with chronic lung disease, reactive airway disease and/or pulmonary hypertension, sedation wean could be associated with agitation leading to bronchospasm and/or pulmonary hypertensive crisis. There is a need to explore a sedative infusion for a smooth extubation trial in children. An ideal sedative for a smooth extubation trial would be a short acting agent, with a quick on-off effect, with no tendency for tolerance or accumulation in lipid stores and minimal side effects. Propofol allows adequate sedation with spontaneous breathing, thereby allowing ventilator weaning around the time of extubation. Since it acts as an intravenous general anesthetic, it assists in keeping the lines and tubes in appropriate positions while allowing ventilator weaning.

Propofol has a black box warning for a prolonged use as an infusion in children. The black box warning relates to propofol infusion syndrome (PRIS), which is a rare but often fatal syndrome, characterized by lactic acidosis, rhabdomyolysis, cardiac failure (bradycardia leading to asystole) associated with propofol infusion over prolonged period of time (13). Though the incidence rate and susceptibility to PRIS in the pediatric population are not known, PRIS is very rare if propofol is used <24 h at <66 mcg/kg/min (~ 4 mg/kg/h) dose with continuous monitoring (14). The data regarding propofol use in ICU and incidence of PRIS has been studied in both pediatric and adult population, but mostly by use of retrospective studies with varying use of PRIS definitions as well as inconsistent reporting of all cases (14). One of those retrospective studies had mentioned incidence of PRIS as high as 33%, with the use of average propofol infusion of 7.27 mg/kg/h (120.6 mcg/kg/min) for an average of 123.3 h (15). Due to this possible risk of PRIS, propofol has not been widely used in general pediatric population in PICU as a sedative agent. Propofol has many unique properties, which makes it very helpful sedation agent like rapid onset of action as well as short duration of action (9).

Keeping its possible life-threatening side effect in mind (PRIS), we aimed at using Propofol as a sedative agent in our PICU population around peri-extubation period for children who are mechanically ventilated and are almost ready for extubation. The ideal peri-extubation sedative agent should be titratable and should not have respiratory and/or hemodynamic depressing effects (16). This sedative agent around extubation should also be short acting without any cumulative effects to allow for spontaneous breathing after its discontinuation (16). Dexmedetomidine, a short acting sedative has been reported to facilitate extubation in critically ill adults (4, 5), but the studies of use of dexmedetomidine as a peri-extubation sedative in children have provided conflicting data regarding its use to facilitate extubation in critically ill children (6, 7). In our cohort of critically ill, patients, mechanically ventilated >48 h and maintained on ≥2 sedative agents, a short-term (<12 h) infusion of propofol was found to be associated with high (97%) extubation success rate. Also, propofol infusion was not associated with any significant hypotension or lactic acidosis. It is possible that we did not see significant lactic acidosis in any of our patients during propofol infusion because median propofol infusion dose and duration were 64.7 mcg/kg/min and 7:53 h respectively. It is important to understand that a large sample size is needed to assess the true burden of PRIS (17). Current literature related to PRIS suggests that elevation in plasma Creatine kinase (CK) occurs before elevation in plasma lactate levels (17, 18). Therefore, it might be valuable to implement a screening protocol, which involves periodic monitoring of CK and lactate levels during propofol infusion.

Our study had several limitations—a retrospective chart review from a single center, a small sample size and lack of any information on extubation success rate following other sedative infusions in similar patient population. Also, due to retrospective nature of the study and due to lack of routine monitoring of depth of sedation (SBS scores) in our unit during the study period, we do not have details of the depth of sedation achieved by propofol infusion. Since both heart rate and blood pressure 4 h before and during propofol infusion (Table 3) were not significantly different, we believe that there was no significant increase in heart rate or blood pressure during propofol infusion and that propofol infusion was associated with adequate depth of sedation in our cohort. Though none of the patients in the study needed any intervention for transient, self-resolving hypotension or developed lactic acidosis, the retrospective nature of the study and small sample size limits our ability to conclude that practice of short-term propofol infusion is safe. A much larger sample size is warranted for accurate safety information of propofol infusion in young patients. Though, based on selection criteria, we are limited in generalizing our results beyond patients who were intubated for >48h, on ≥2 sedative infusions, who received propofol for 4–24 h and who were extubated, we believe that the cohort described in the study is representative of challenging patients who we come across in the PICU and who could benefit from a sedative agent such as propofol as a bridge to successful extubation. Extubation success rate was found to be high (34/35 patients) in our study, suggesting high efficacy of propofol as a bridge to extubation. Due to retrospective nature of the study and due to assessment of propofol alone as a peri-extubation sedative, we are unable to conclude that propofol infusion is more efficacious than any other approach to extubation.

**Table 3:** Details of hemodynamic parameters before, during and after propofol infusion (*N* = 35).

**Hemodynamic parameters**		***p-value***
HR^*^ 4 h before and during infusion	102 (85.25-122); 102 (85.25-122)	NS
MAP^**^ 4 h before and during infusion	66.33 (59.08-77.67); 63.83 (55-70)	NS
HR during and 4 h after infusion	102 (85.25-122; 127 (105.5-144.5)	NS
MAP during and 4 h after infusion	63.83 (55-70);72.3 (65-82.75)	NS
HR 4hrs before and 4 h after infusion	102 (85.25-122);127 (105.5-144.5)	NS
MAP 4hrs before and 4 h after infusion	66.33 (59.08-77.67);72.3 (65-82.75)	NS

* *HR, Heart Rate (per minute)*,

** *MAP, Mean Arterial Pressure (mm of Hg), p-values obtained using Wilcoxon Rank Sum Test, significant p-value ≤ 0.05*.

## Conclusions

In a small cohort of critically ill children, mechanically ventilated for >48 h and maintained on ≥2 sedation infusions in our mixed pediatric intensive care unit, propofol infusion used between 4 and 24 h was found to be a feasible bridge to extubation. An appropriate next step would be to conduct larger, multicenter, prospective studies on propofol infusion as a bridge to extubation in critically ill children supported on mechanical ventilation.

## Data Availability Statement

The raw data supporting the conclusions of this article will be made available by the authors, without undue reservation.

## Ethics Statement

The studies involving human participants were reviewed and approved by Baylor College of Medicine Institutional Review Board. Written informed consent from the participants' legal guardian/next of kin was not required to participate in this study in accordance with the national legislation and the institutional requirements.

## Author Contributions

UB, AP, MT, MS, and EA contributed to design of the study, data gathering, data analysis, writing and editing the manuscript.

## Conflict of Interest

The authors declare that the research was conducted in the absence of any commercial or financial relationships that could be construed as a potential conflict of interest.
